# Line-Scan Defect Detection for Printed Laminated Films Using Multi-Template Matching and Residual-Driven Column-Band Compensation

**DOI:** 10.3390/s26206372

**Published:** 2026-10-09

**Authors:** Pengjian Zhang, Wen Ren

**Affiliations:** 1 College of Mechanical and Electrical Engineering, Fujian Agriculture and Forestry University, Fuzhou 350002, China; 52412048001@fafu.edu.cn; 2 School of Mechanical and Electrical Engineering, Sanming University, Sanming 365004, China

**Keywords:** printed laminated films, defect detection, line-scan imaging, multi-template matching, deformation compensation

## Abstract

Machine vision enables efficient, non-contact industrial quality inspection. Online inspection of printed laminated films is challenging because complex graphics and transport-induced stretching cause appearance variations and spatially nonuniform mismatches. Global registration cannot fully correct these mismatches, whereas unrestricted dense deformation may absorb small defects. We propose an interpretable system based on a qualified multi-template library and residual-driven column-band compensation. Low-resolution retrieval selects geometrically compatible references; intermediate-resolution ORB–RANSAC registration and original-resolution warping then establish global correspondence. Compensation is activated only in residual-selected bands and constrained to the web-motion direction. Template-tolerant multi-scale Frangi comparison and component-level multi-template voting generate the final defect mask. The independent test set comprised 1500 production-line images: 200 defect-bearing images containing 823 annotated defect instances and 1300 defect-free images. Under the defect-instance-level evaluation, the system produced 765 true positives, 58 false negatives, and 31 false-positive predicted components, corresponding to 92.95% recall and 96.11% precision. The mean processing time was 357.06 ms/image. The results indicate that the method can balance residual-mismatch suppression and defect preservation under the evaluated production conditions.

## 1. Introduction

Automated visual inspection has become an important component of industrial quality control because it enables non-contact, repeatable, and high-throughput evaluation of product appearance. Machine-vision systems have consequently been applied to defect detection in mechanical and metal-product manufacturing, printing, textiles, and other industrial sectors [1,2,3]. Within this broad field, online inspection of printing and continuous-web products remains challenging because defects occur against intentional graphics composed of text, edges, color transitions, and fine decorative structures. Small ink spots, missing print, scratches, streak-like defects, and local misregistration may therefore resemble valid artwork elements [4,5,6,7,8,9]. The problem is further complicated when printed laminated film is acquired by a line-scan camera during continuous roll-to-roll transport. Although frame triggering, line triggering, and web guiding stabilize image formation, illumination variation, tension fluctuation, local stretching, and residual motion can still alter the appearance and geometry of otherwise qualified products. Reliable inspection must therefore separate true printing defects from both normal graphic content and production-induced appearance variations, which makes the choice of detection route a central design decision.

Existing visual defect-detection methods can be broadly divided into deterministic image-processing methods and learning-based methods according to how defect evidence is represented and obtained [1,2]. Deterministic methods judge abnormalities using explicitly designed cues, such as intensity differences, edges, textures, structural features, or deviations from a qualified reference image. Their processing rules and intermediate results are directly observable, allowing the decision process to be inspected and the relevant thresholds or constraints to be adjusted for a specific production task. Learning-based methods instead learn discriminative representations from data and have been applied to candidate-region filtering for printing defects and small-defect detection in packaging prints [9,10]. When the training data adequately cover the expected operating conditions, these methods can reduce reliance on manually designed features and tolerate certain appearance and positional variations without requiring strict pixel-wise comparison with a reference image. However, obtaining such coverage is not straightforward in industrial inspection. Supervised methods require representative labeled anomalies, whereas real defects are often scarce, costly to annotate, and impossible to enumerate completely before deployment [11]. Unsupervised anomaly detection reduces the need for defect labels by learning from normal samples, but it still relies on the normal appearance distribution of a particular product or category; accordingly, many established methods train separate models for different object categories [12]. The operating data distribution may also change after deployment. Variations in illumination, object pose, or background appearance can create a mismatch between training and test data, thereby degrading anomaly-detection performance [13]. Data efficiency and rapid adaptation across products therefore remain important challenges for industrial anomaly detection [14]. These difficulties become more pronounced on production lines with many product variants or frequent product updates; a change in printed design, material batch, imaging parameters, or equipment condition may alter the observed data distribution, making additional data collection, model adaptation, and repeated validation necessary. Maintaining several product-specific models further increases deployment and management effort. Overall, deep learning provides an effective tool for industrial inspection, although its stable deployment generally requires representative data, appropriate updating, and validation as products and production conditions change. When qualified samples of a new product can be obtained after changeover, updating a qualified reference library does not require waiting for representative defects or retraining a detection model. Deterministic comparison with such reference images therefore remains a practical complementary route. Its reliability, however, depends on two consecutive prerequisites: the selected reference must represent the current normal appearance, and the inspected image must be brought into accurate correspondence with that reference.

The first prerequisite is reference representativeness. Early reference-based printing inspection commonly used one defect-free golden master [5], but a fixed image cannot cover all permissible variations observed during long-term continuous production. If the reference differs from a qualified inspection image in illumination, material state, or local printed appearance, the resulting normal discrepancy enters the subsequent registration and defect-analysis stages and may be reported as an anomaly. Previous studies have increased reference tolerance in different ways. Zhang et al. [6] constructed bright and dark tolerance templates to accommodate limited positional and intensity variation. Liu et al. [7] registered several qualified images, divided them into sub-blocks, extracted Canny edges, and fused the resulting information into a multi-template edge representation. Li et al. combined registered RGB sub-images, masks, candidate extraction, and twice gradient matching to suppress artifacts caused by local deformation [8]. These strategies improve tolerance to normal variations, but they either retain one reference with an enlarged acceptance range or compress information from several qualified images into a composite representation. The latter changes the complete gray level and spatial context of each original reference and may make agreement or disagreement with an individual normal sample less observable. A more direct alternative is to maintain multiple qualified images as separate complete references and retrieve a geometrically compatible subset for each inspection image. Such a template library uses readily available qualified products to represent permissible production variation without merging their individual image content. Nevertheless, selecting a suitable reference only determines what the inspection image should resemble; determining whether the two images differ because of a defect still requires accurate geometric correspondence.

The second prerequisite is registration accuracy. For global mismatch in printing images, Shankar et al. [5] developed a real-time web-printing inspection system based on comparison with a defect-free golden master. Zhang et al. [6] determined the first row of each captured image by one-dimensional projection and then performed registration using a shape-context descriptor that incorporates contour concavity and convexity. Liu et al. [15] further refined a scale-adaptive localized matching region through affine alignment based on the enhanced correlation coefficient. These methods can effectively compensate for global positional and affine deviations, but a single transformation shared by the entire image cannot describe spatially nonuniform residuals. To improve local correspondence, Chen et al. [16] proposed an automatic feature-region searching method that selects discriminative shape regions for registration. Chen et al. [17] subsequently combined SIFT-based global registration with Active Demons deformation optimization to correct both rigid and elastic deformation. Kroeger et al. [18] developed Dense Inverse Search (DIS) optical flow, which estimates pixel-wise two-dimensional motion efficiently through inverse search and provides a general mechanism for correcting spatially varying displacement. Li et al. [19] proposed APPR-Net, which obtains coarse alignment through a four-stream affine module and progressively refines a dense deformation field using a multi-scale pyramid network. Although these local and dense methods enlarge the correctable deformation range, increasing deformation freedom also increases the need for structural constraints. In defect inspection, an excessively flexible deformation field may interpret a localized anomaly as geometric displacement and thereby attenuate its response. This trade-off is particularly relevant to line-scan imaging of moving flexible webs. Gafurov et al. [20] analyzed the effects of web tension and other process factors on roll-to-roll registration errors and combined passive and active compensation. Lee et al. [21] proposed defect-aware register sensing and alignment correction under varying web-tension conditions. For line-scan images, Feng et al. [22] formulated horizontal registration as a one-dimensional disparity-estimation problem to compensate for spatially varying distortion associated with motion fluctuation. These studies demonstrate the strong process dependence and directionality of residual mismatch in moving-web imaging. On practical web-production lines using line-scan cameras, residual mismatch after global registration is often concentrated in limited column bands, and the dominant displacement follows the web-motion direction. A single global transform is therefore insufficient, whereas a general two-dimensional dense warp provides more freedom than the observed deformation requires. The remaining need is a residual-triggered local correction that acts only where systematic mismatch is present, follows the dominant physical direction, and leaves unrelated regions unchanged.

To satisfy these two prerequisites jointly, this study proposes a multi-template matching and column-band compensation method for the online inspection of printed laminated films. Unlike the multi-template edge-fusion strategy in ref. [7], which merges multiple qualified images into a composite representation, the proposed method retains the selected images as independent complete references, processes each reference branch separately, and then aggregates the defect decisions across branches. Compared to dense-deformation methods, such as Active Demons [17] and DIS [18], local compensation is activated only within residual-selected column bands, the displacement is restricted to a bounded one-dimensional search along the web-motion direction, and a correction is accepted only when the residual improvement reaches the specified threshold. This direction-constrained design is consistent with the horizontal-disparity principle in ref. [22], while further introducing residual-triggered band localization, a correction-acceptance mechanism, and cross-template consensus specifically for defect inspection. By limiting both the spatial support and the degrees of freedom of local correction, the proposed method aims to suppress pseudo-defects caused by residual misregistration while reducing the risk of attenuating true defect evidence.

The deformation model is intended for cases in which global registration removes the dominant overall pose difference and the remaining mismatch is concentrated in a limited number of column bands, dominated by displacement along the known web-motion direction, approximately consistent along the orthogonal direction, and contained within the preset search range. It is not designed for arbitrary two-dimensional deformation, deformation dominated by orthogonal displacement, displacement beyond the search range, or deformation that varies strongly along the orthogonal direction. The residual signal can localize regions that may require geometric correction but cannot directly distinguish local deformation from a genuine defect. Because the column residual is averaged over the full image height, localized defect is generally unlikely to activate a continuous compensation band by itself. Nevertheless, when a defect lies inside a band activated primarily by deformation, defect-response attenuation cannot be completely excluded. Independently processing multiple complete templates also increases computational cost, and the current fixed operating parameters do not adapt automatically to changes in production conditions. On the evaluated production-line dataset, the proposed method achieved high defect-instance recall and preserved defect responses more effectively than the compared dense-deformation methods. Its processing capacity supports the normal acquisition rate evaluated in this study, whereas sustained operation at the maximum acquisition rate requires further acceleration or increased processing concurrency.

## 2. Materials and Methods

This section describes the online inspection platform and image-processing pipeline for printed laminated films. To address spatially nonuniform mismatch caused by web motion and local stretching, multi-template registration establishes overall correspondence between each inspected image and qualified reference images, and the registration residual identifies the column bands requiring correction. Local compensation is confined to these bands. Pixels outside the selected bands retain their globally registered values. Template-tolerant structural-response comparison then generates defect candidates, which are consolidated by multi-template voting into the final defect mask. By limiting the spatial extent and degrees of freedom of local correction, the pipeline reduces residual disagreement around normal printed structures and lowers the risk of attenuating true defect responses.

### 2.1. Line-Scan Inspection System

#### System Composition

The inspection system consists of a roll-to-roll transport unit, a line-scan imaging unit, trigger and motion-sensing components, an edge-guiding device, and an industrial computer. The principal components and their functions are summarized in Table 1. The imaging unit is installed above the inspection region, while line-light units illuminate the film from complementary directions to reduce shadows and reflection-dependent intensity variation. The edge-guiding device stabilizes the lateral position of the moving web before it reaches the imaging region. Images acquired by the line-scan camera are transferred to the industrial computer for template retrieval, registration, local compensation, defect analysis, visualization, and result storage.

**Figure 1:**
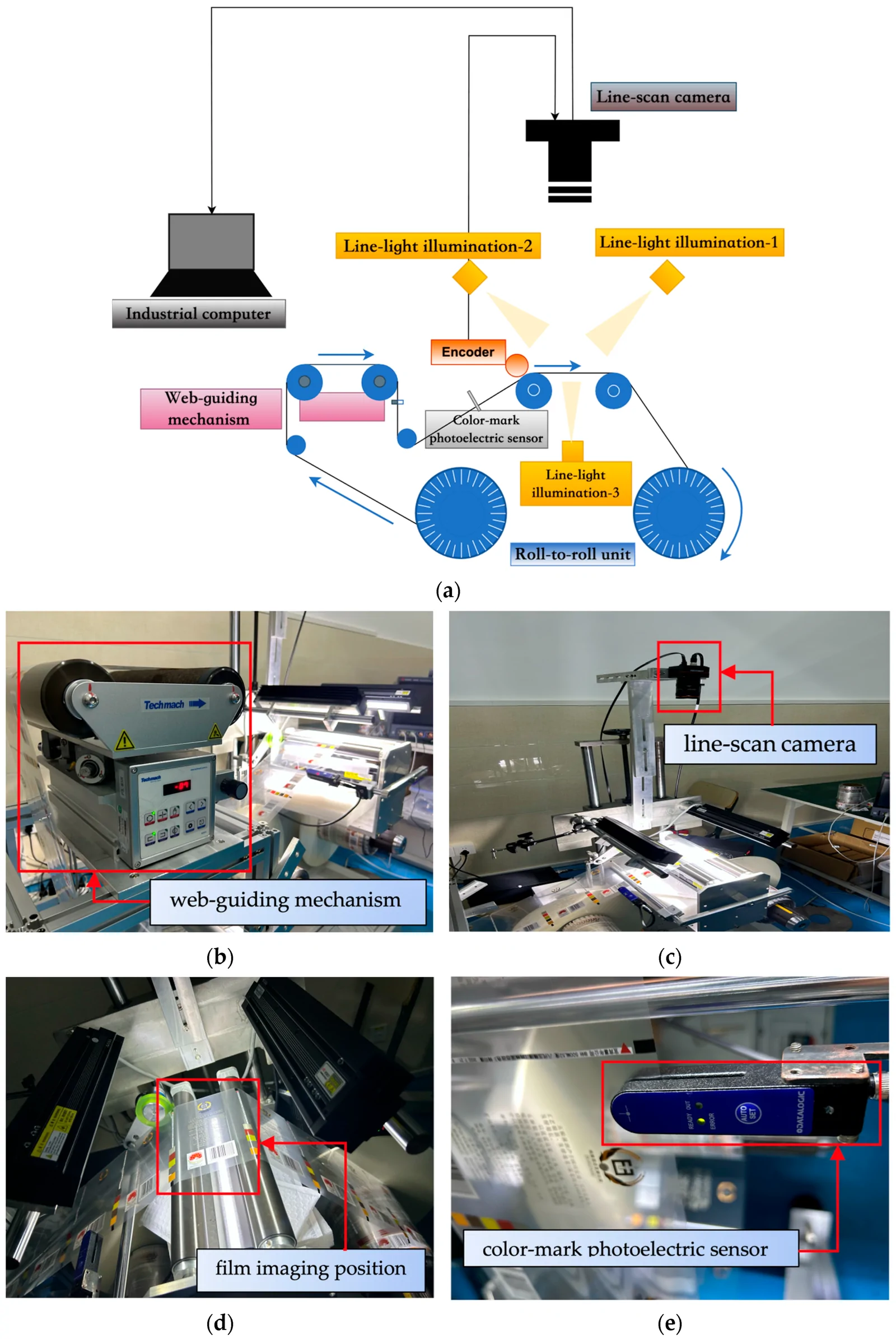
Image-acquisition platform: (**a**) transport and imaging principle; (**b**) web-guiding mechanism; (**c**) line-scan camera position; (**d**) film imaging position; and (**e**) color-mark photoelectric sensor.

Figure 1 links the system schematic to the physical acquisition platform. Figure 1a shows the web-transport path and the arrangement of the line-scan camera, illumination units, encoder, color-mark sensor, and industrial computer. Figure 1c,d show the imaging region and camera installation, and Figure 1b presents the web-guiding mechanism used to suppress lateral wandering. The color-mark sensor in Figure 1e detects the registration mark of each repeated printed unit and issues the corresponding frame-trigger pulse. During image acquisition, the encoder converts roller motion into line-trigger pulses, allowing each camera line to be acquired according to the measured web displacement. Frame and line triggering maintain correspondence between the repeated artwork and image boundaries and reduce geometric variation caused by speed fluctuations.

### 2.2. Overall Framework

**Figure 2:**
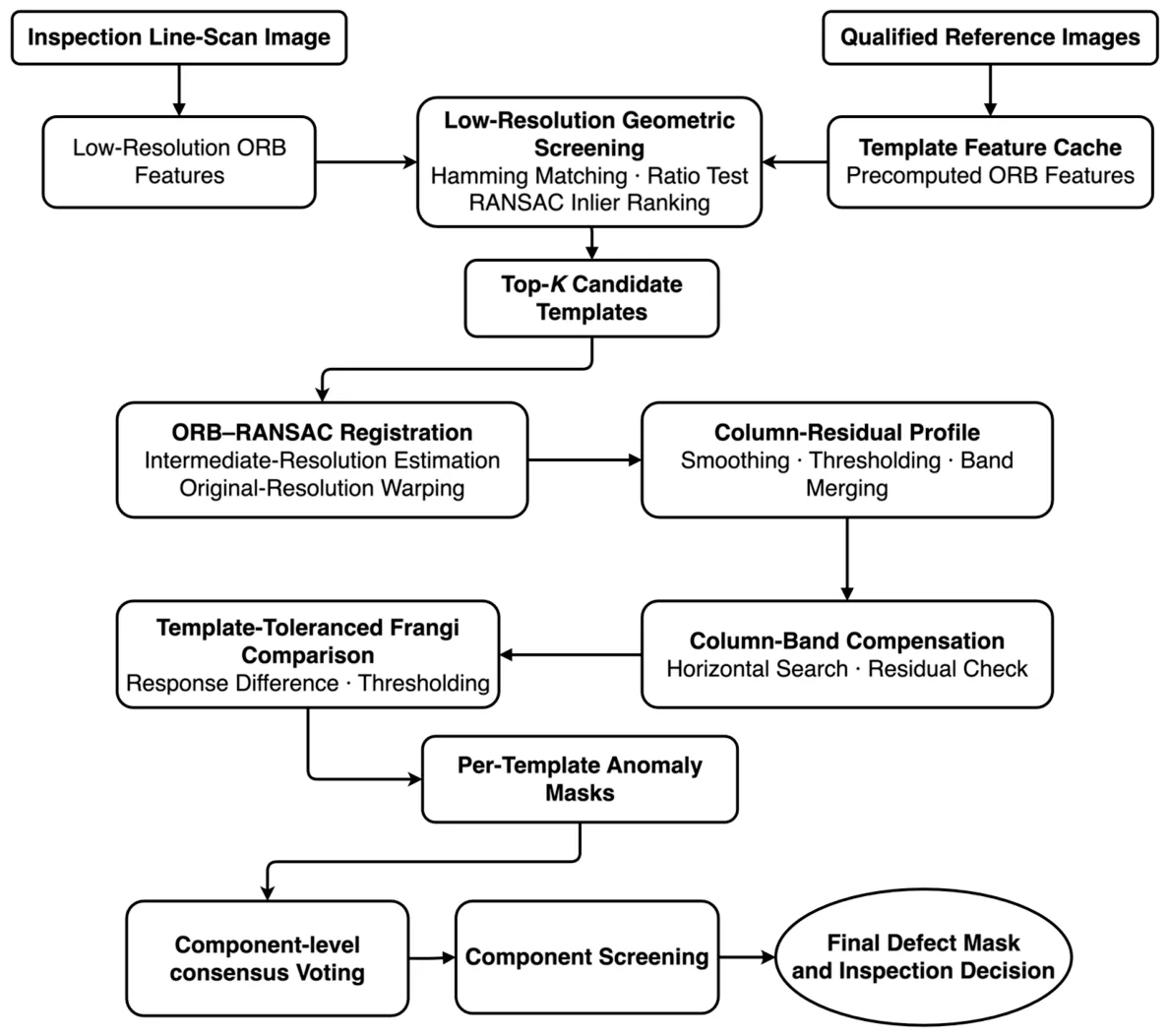
Overall pipeline of the proposed method: low-resolution geometric screening, per-template global registration and column-band compensation, template-tolerant Frangi comparison, component-level consensus voting, and final component screening.

As illustrated in Figure 2, the proposed framework first evaluates all templates in the normal template library at a reduced resolution using Oriented FAST and Rotated BRIEF (ORB) features. Following the coarse-to-fine principle of image pyramids [23], the top *K* templates are retained according to the inlier support obtained using random sample consensus (RANSAC). Each retained template then undergoes intermediate-resolution ORB–RANSAC registration, original-resolution warping, and residual-triggered column-band compensation. Template-tolerant Frangi comparison generates a structural-anomaly mask for each template branch. Finally, component-level consensus voting and connected-component screening produce the defect-detection result.

### 2.3. Coarse-to-Fine Multi-Template Registration

#### 2.3.1. Template Library and Feature Cache

Normal appearance can vary across continuous production, making a single reference image insufficient. Let $X={\left\{X_{\ell }\right\}}_{\ell =1}^{M}$ denote a pool of *M* qualified defect-free images acquired under representative operating conditions. From this pool, we construct the normal template library $T={\left\{T_{i}\right\}}_{i=1}^{N}$, where *T_i_* is the *i*-th selected template, *N* is the number of templates, and the inspected image is denoted by *I*. To construct a representative library while limiting redundancy, we employ an ORB–RANSAC-based farthest-first selection strategy. Feature matching and geometric verification use the low-resolution ORB–RANSAC procedure; further details are provided in Section 2.3.2. During template-library selection, this procedure is used to measure the similarity between qualified reference candidates. Let 𝒯*_m_* ⊂ 𝒳 denote the partial template library after *m* selection iterations. Starting from a reproducibly fixed initial template, each unselected candidate *X_ℓ_* ∉ 𝒯*_m_* is compared with every selected template *T_i_* ∈ 𝒯*_m_*. Let *n^g^*(*X_ℓ_*,*T_i_*) denote their low-resolution RANSAC inlier count. The similarity score of *X_ℓ_* with respect to the current library is defined as follows:

(1) $$s_{m}\left(X_{\ell }\right)=\underset{T_{i}\in T_{m}}{\max}n^{g}\left(X_{\ell },T_{i}\right).$$

The next template is selected according to the following equation:

(2) $$\ell^{*}=\underset{\ell :\;X_{\ell }\notin T_{m}}{\arg\min}\;s_{m}\left(X_{\ell }\right),\;T_{m+1}=T_{m}\cup \left\{X_{\ell^{*}}\right\}.$$

Thus, each candidate is evaluated against the entire current library, and the candidate that remains least similar even to its most similar selected template is added next. This procedure preferentially supplements feature-geometric states that are insufficiently represented by the current library, while candidates already well represented by an existing template receive lower selection priority. The process is repeated until the selected-candidate score *s_m_*(*X_ℓ_*) approaches stability.

For each template, low-resolution and intermediate-resolution ORB features are precomputed and stored in a lightweight feature cache for geometric screening and refined registration, respectively. The original template images are retained, while preprocessing and Frangi-response computation are performed only for the retrieved candidates when required. For each inspected image, the corresponding ORB features are extracted once at each scale and reused across template comparisons.

#### 2.3.2. Low-Resolution Geometric Screening

**Figure 3:**
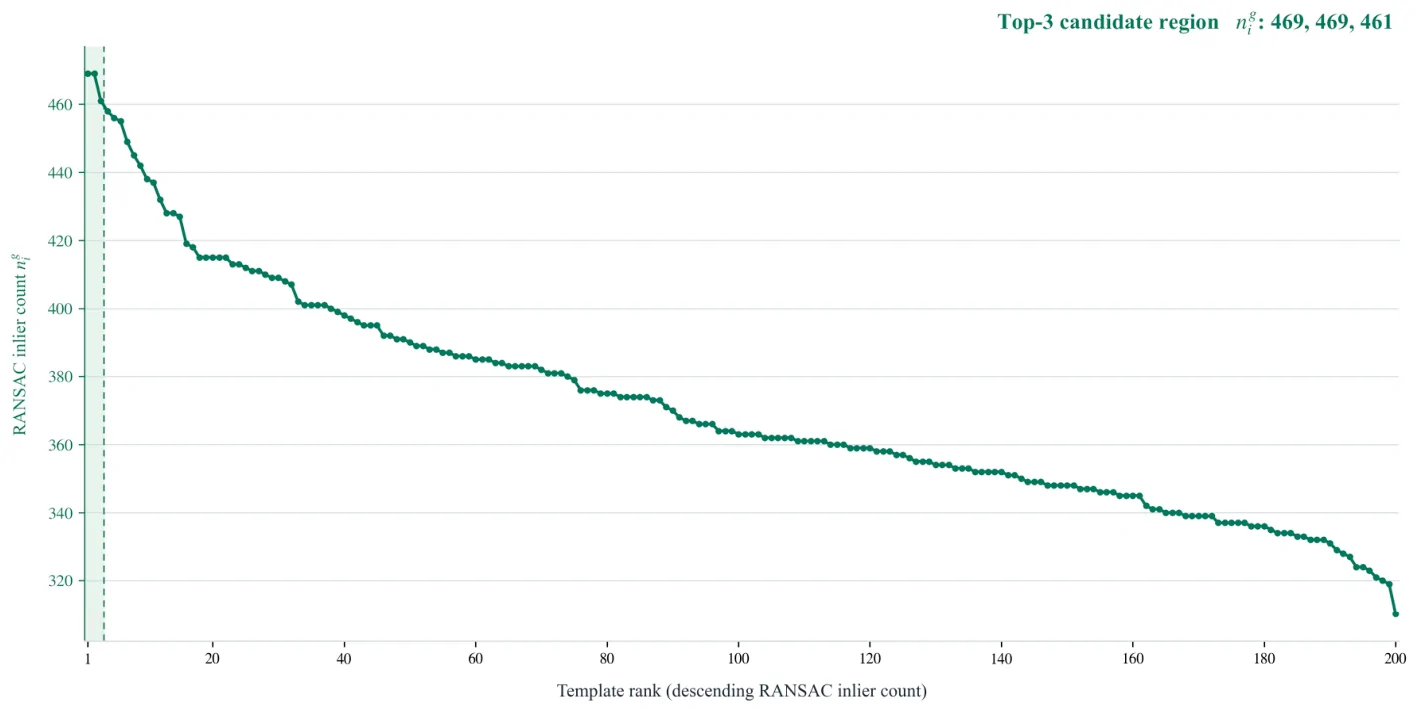
Representative result of low-resolution geometric screening. The 200 templates are ranked in descending order of the RANSAC inlier count $n_{i}^{g}$, and the shaded region indicates the retained Top-3 candidate set.

Running full detection against every template would make the computational cost grow with the library size. The method therefore first evaluates global geometric consistency at a reduced resolution. Let *I^g^* and $T_{i}^{g}$ denote the low-resolution versions of *I* and *T_i_*, respectively. Oriented FAST and Rotated BRIEF (ORB) keypoints and binary descriptors are extracted from these images [24]. Descriptor pairs are obtained using Hamming distance and filtered by the nearest-to-second-nearest distance ratio [25]. Random sample consensus (RANSAC) then estimates a homography from the retained correspondences and rejects inconsistent matches [26]. For a matched point pair, the projective relation is expressed as follows:

(3) $$\lambda_{j}q_{j}=H_{i}^{g}p_{j}$$

where $p_{j}={\left[x_{j},y_{j},1\right]}^{T}$ and $q_{j}={\left[u_{j},v_{j},1\right]}^{T}$ are homogeneous coordinates in the inspected image and the template, respectively; and *λ_j_* ≠ 0 is the homogeneous scale factor. Let *z_ij_* ∈ {0, 1} indicate whether the *j*-th retained correspondence is a RANSAC inlier. Consistent with the notation introduced in Section 2.3.1, the geometric support of template *T_i_* is $n_{i}^{g}=n^{g}\left(I,T_{i}\right)$ and is computed as follows:

(4) $$n_{i}^{g}=\sum_{j=1}^{m_{i}^{g}}z_{\mathrm{ij}},$$

where $m_{i}^{g}$ is the number of correspondences retained by the Lowe ratio test. Templates for which homography estimation fails are removed. The remaining templates are sorted by $n_{i}^{g}$ in descending order, and the first *K* templates form the candidate set:

(5) $$C_{K}={\mathrm{TopK}}_{i\in V}\left(n_{i}^{g}\right),$$

where TopK selects the indices of the *K* largest scores, *K* is the number of retained candidates, and 𝒱 is the set of templates with valid homographies. The retained candidates are then passed to the refined registration stage. Figure 3 shows the low-resolution geometric-screening results for a representative inspected image. Each point represents one template, and the templates are arranged from left to right in descending order of their RANSAC inlier counts. The horizontal coordinate denotes the rank after sorting rather than the original template index, whereas the vertical coordinate denotes the number of RANSAC inliers obtained between the inspected image and the template at that rank. A larger inlier count indicates stronger geometric compatibility. The shaded region contains ranks 1–3, which form the candidate set 𝒞_3_ and are passed to the refined registration stage.

#### 2.3.3. Refined Global Registration of Retained Candidates

The reduced screening image preserves the layout but contains limited detail. ORB matching and RANSAC estimation are therefore repeated for the retained candidates at an intermediate resolution. Let $H_{i}^{a}$ be the homography estimated at that scale and let *S_I_* and *S_T_* map the original inspected image and template to their corresponding alignment scales. The homography used to warp the original image is expressed as follows:

(6) $$H_{i}=S_{T}^{-1}H_{i}^{a}S_{I}$$

Applying *H_i_* to the original image yields the globally registered result ${\tilde{I}}_{i}$. This formulation estimates geometry efficiently on smaller images while preserving the original resolution for subsequent comparison. The resulting image is then evaluated by the residual analysis described next.

### 2.4. Residual-Triggered Constrained Column-Band Compensation

#### 2.4.1. Column-Band Localization

Line-scan images are assembled from scanning lines acquired continuously during web transport. As noted above, local stretching of the flexible web or variations in sampling intervals can produce different displacements across column bands, causing the geometric deviation to vary with image position. A homography applies a single projective transformation to the entire image; it can correct the dominant global geometric deviation but cannot simultaneously eliminate local mismatch among the column bands. Therefore, after global registration, the column residual is used to locate and compensate the remaining local mismatch. For template *T_i_* and its globally registered image ${\tilde{I}}_{i}$, the absolute residual *D_i_* and column profile *p_i_* are defined as follows:

(7) $$D_{i}\left(x,y\right)=\left|T_{i}\left(x,y\right)-{\tilde{I}}_{i}\left(x,y\right)\right|$$

(8) $$p_{i}\left(x\right)=\frac{1}{h}\sum_{y=1}^{h}D_{i}\left(x,y\right)$$

where *x* and *y* denote the image column and row, respectively; and *h* is the image height. The profile is smoothed by a one-dimensional averaging filter to obtain ${\bar{p}}_{i}\left(x\right)$. A single localization threshold is then calculated as follows:

*τ_i_* = max(*τ*_0_,*μ_i_* + *κσ_i_*), (9)

where *μ_i_* and *σ_i_* are the mean and standard deviation of ${\bar{p}}_{i}$, respectively; *τ*_0_ is a minimum residual threshold; and *κ* controls the sensitivity to image-specific residual variation. Consecutive columns satisfying ${\bar{p}}_{i}\left(x\right)>\tau_{i}$ form core intervals. Each interval is enlarged by a fixed margin *w_p_*, and overlapping intervals are merged to obtain the candidate bands *B_ij_*, where *j* indexes the bands associated with template *T_i_*. Numerical values are reported in Section 3.

#### 2.4.2. Constrained Displacement Search and Acceptance Criterion

For each candidate band *B_ij_* = [*x*_0_, *x*_1_), where *x*_0_ and *x*_1_ are its left and right boundaries, respectively, control points *x_c_* are placed along the column direction. Within the local window Ω*_c_*, the horizontal displacement is selected from a bounded range by minimizing the mean absolute residual:

(10) $$d_{\mathrm{ij}}\left(x_{c}\right)=\underset{u\in \left[-d_{\max},d_{\max}\right]}{\arg\min}\frac{1}{\left|\Omega_{c}\right|}\sum_{\left(x,y\right)\in \Omega_{c}}\left|T_{i}\left(x,y\right)-{\tilde{I}}_{i}\left(x-u,y\right)\right|$$

where *u* is a candidate horizontal displacement, *d*_max_ is the maximum permitted displacement, and |Ω*_c_*| is the number of pixels in the local window. The discrete estimates are interpolated and smoothed to obtain *d_ij_*(*x*). The candidate-corrected image within *B_ij_* is denoted by $I_{\mathrm{ij}}^{\prime }$ and changes only the horizontal sampling coordinate:

(11) $$I_{\mathrm{ij}}^{\prime }\left(x,y\right)={\tilde{I}}_{i}\left(x-d_{\mathrm{ij}}\left(x\right),y\right),\;x\in B_{\mathrm{ij}}$$

According to Equation (7), the mean residuals within *B_ij_* before and after the candidate correction are calculated and denoted by $E_{\mathrm{ij}}^{0}$ and $E_{\mathrm{ij}}^{1}$, respectively. The relative improvement is given by:

(12) $$r_{\mathrm{ij}}=\frac{E_{\mathrm{ij}}^{0}-E_{\mathrm{ij}}^{1}}{E_{\mathrm{ij}}^{0}+\varepsilon }$$

where *ε* is a small positive constant and *τ_r_* is the minimum relative improvement required to accept the correction. For an accepted band, a taper *ω_ij_*(*x*) ∈ [0, 1] blends the corrected and globally registered images; otherwise, the globally registered pixels are retained. The final compensated image for template branch *i* is denoted by $I_{i}^{c}$:

(13) $$I_{i}^{c}\left(x,y\right)=\{\begin{array}{cc} \omega_{\mathrm{ij}}\left(x\right)I_{\mathrm{ij}}^{\prime }\left(x,y\right)+\left[1-\omega_{\mathrm{ij}}\left(x\right)\right]{\tilde{I}}_{i}\left(x,y\right), & x\in B_{\mathrm{ij}},r_{\mathrm{ij}}\geq \tau_{r},\\ {\tilde{I}}_{i}\left(x,y\right), & \mathrm{otherwise}. \end{array}$$

**Figure 4:**
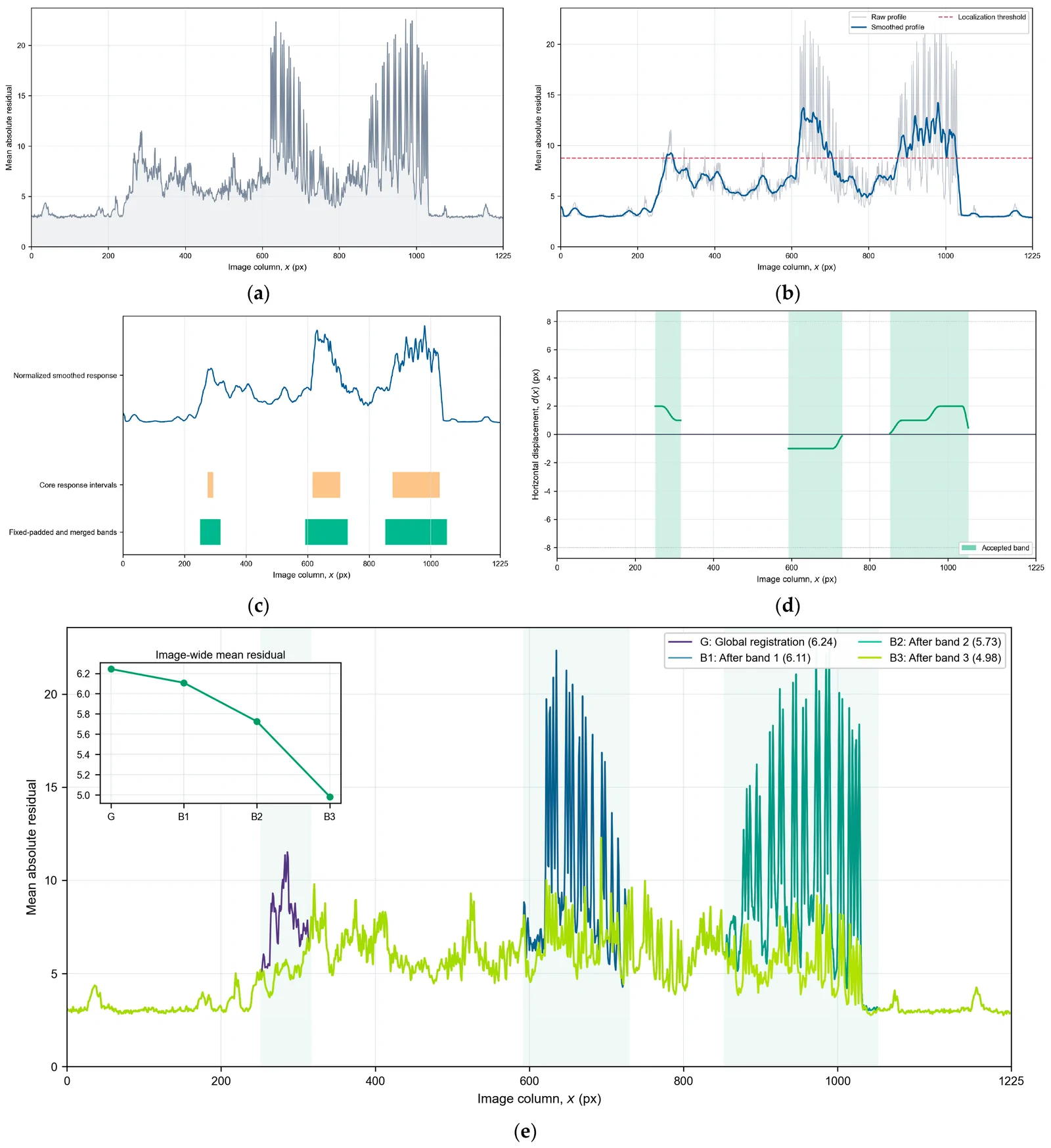
Evolution of the column-residual profile: (**a**) raw profile; (**b**) smoothed profile and localization threshold; (**c**) core intervals and the bands obtained after fixed padding and merging; (**d**) horizontal displacement curves; and (**e**) profile changes after sequentially accepted compensation.

This design constrains local correction by residual support, displacement direction, search range, and measured improvement. Figure 4 shows how the column-residual profile evolves from localization to sequential correction. The resulting image $I_{i}^{c}$ provides the geometrically stabilized input for the subsequent structure-aware comparison. All candidate bands are determined once from the initial post-registration residual profile and processed sequentially without reselection. Figure 4e shows only the resulting profile evolution.

### 2.5. Structure-Aware Defect Response

#### 2.5.1. Multi-Scale Structure Enhancement

Direct grayscale comparison is sensitive to illumination variations and small remaining edge mismatches. The registered template and compensated inspected image are therefore processed identically using local contrast enhancement and edge-preserving denoising. A multi-scale Frangi filter is then used to characterize local second-order structural responses [27]. At scale *σ*, the Hessian matrix of an image *G* is given by:

(14) $$H_{\sigma }\left(x,y\right)=\left[\begin{array}{cc} G_{\mathrm{xx}}^{\sigma }\left(x,y\right) & G_{\mathrm{xy}}^{\sigma }\left(x,y\right)\\ G_{\mathrm{xy}}^{\sigma }\left(x,y\right) & G_{\mathrm{yy}}^{\sigma }\left(x,y\right) \end{array}\right],$$

where $G_{\mathrm{xx}}^{\sigma }$ and $G_{\mathrm{yy}}^{\sigma }$ are the scale-normalized second derivatives along the two image axes, and $G_{\mathrm{xy}}^{\sigma }$ is the corresponding mixed derivative. Let *λ*_1_ and *λ*_2_ be the eigenvalues ordered algebraically such that *λ*_1_ ≥ *λ*_2_. Responses are computed at several scales, and the maximum response is retained:

(15) $$R_{b}=\frac{\left|\lambda_{2}\right|}{\left|\lambda_{1}\right|+\epsilon }$$

(16) $$S=\sqrt{\lambda_{1}^{2}+\lambda_{2}^{2}}$$

(17) $$V\left(x,y\right)=\underset{\sigma \in \Sigma }{\max}\left\{\exp\left(-\frac{R_{b}^{2}}{2\beta^{2}}\right)\left[1-\exp\left(-\frac{S^{2}}{2c^{2}}\right)\right]\right\}$$

Here, *R_b_* describes the relative relationship between the two principal curvatures and characterizes the local shape anisotropy: it is small when one directional curvature dominates and increases as the two curvature magnitudes become comparable. The quantity *S* measures the overall magnitude of the Hessian response and therefore the strength of the local second-order structure; it is small in flat regions and increases at pronounced structural changes. Consequently, *β* controls the shape selectivity, whereas *c* suppresses flat regions and weak background responses. Only responses satisfying *λ*_1_ > 0 are retained to select dark structures on a bright background. The set Σ contains the evaluated scales, and *ε_F_* > 0 is a small stabilizing constant; the numerical settings of Σ, *β*, and *c* are reported in Section 3.

#### 2.5.2. Bidirectional Frangi Comparison with Template-Derived Bounds

Normal text strokes, artwork boundaries, and barcode lines also produce strong Frangi responses in defect-free samples. The template response is therefore used to define the normal response range. For the *i*th template branch, let *V_T_*(*x*,*y*) and *V_I_*(*x*,*y*) denote the normalized multiscale Frangi responses of template *T_i_* and the corresponding compensated inspected image $I_{i}^{c}$, respectively. The upper and lower bounds are constructed only from the template response:

(18) $$\begin{array}{c} U_{T}\left(x,y\right)=\min\left\{1,\;\alpha \mathrm{Dilate}\left(V_{T}\right)\left(x,y\right)+\delta \right\},\\ L_{T}\left(x,y\right)=\max\left\{0,\;\frac{\mathrm{Erode}\left(V_{T}\right)\left(x,y\right)}{\alpha }-\delta \right\}. \end{array}$$

where *U_T_* and *L_T_* are the upper and lower template bounds, respectively. The parameter *α* ≥ 1 provides multiplicative tolerance, while *δ* ≥ 0 provides an additive response margin. The operators Dilate and Erode use the same structuring element. Thus, the bounds allow limited spatial displacement and response variation while retaining the template response within the interval. An inspected response inside this interval is regarded as normal; departures from either side give the anomaly response:

*A_i_*(*x*,*y*) = max{*V_I_*(*x*,*y*) − *U_T_*(*x*,*y*), *L_T_*(*x*,*y*) − *V_I_*(*x*,*y*), 0}. (19)

The first term responds to structures that appear or become stronger in the inspected image, whereas the second responds to structures that are missing or weaker. Morphological operations are applied only to the template response; the inspected response is not expanded. The structuring-element size controls the trade-off between residual-displacement tolerance and sensitivity to small structural changes.

Let Ω*_i_* denote the common valid region of *T_i_* and $I_{i}^{c}$ after excluding a boundary band equal to the structuring-element radius. The candidate mask for the *i*th template is obtained by thresholding *A_i_* within this region:

(20) $$B_{i}\left(x,y\right)=\{\begin{array}{cc} 1, & \left(x,y\right)\in \Omega_{i}\;\mathrm{and}\;A_{i}\left(x,y\right)>\tau_{F},\\ 0, & \mathrm{otherwise}. \end{array},$$

where *τ_F_* is the response threshold. Connected components are then extracted from the binary mask *B_i_*.

### 2.6. Consensus Voting Across the Top − K Templates

Even after global registration and local compensation, a single template may still produce occasional responses because it represents only one normal appearance state. Therefore, the retained *K* template branches generate candidate masks independently and combine them through multi-template voting. The highest-ranked template *T_r_* is used as the reference coordinate system. The candidate mask *B_k_* of branch *k* is mapped to this reference grid using the global homography:

(21) $${\bar{B}}_{k}={\mathrm{Warp}}_{\mathrm{NN}}\left(B_{k},H_{r}H_{k}^{-1}\right),k=1,\ldots ,K,$$

Here, $H_{k}^{-1}$ first maps a point from the coordinate system of template *T_k_* back to the inspected-image coordinate system, and *H_r_* then maps that point to the coordinate system of the reference template *T_r_*. Therefore, the composite transformation $H_{r}H_{k}^{-1}$ transfers the candidate mask from the grid of *T_k_* to the grid of *T_r_*. Nearest-neighbor interpolation is used to preserve the binary mask values.

Because column-band compensation is estimated independently for each branch, candidate regions corresponding to the same defect may have a small horizontal offset after the global mask transformation. To tolerate this bounded inconsistency, voting is performed at the connected-component level. Connected components are extracted from each transformed mask. For a component *C_r_* in the reference branch and a component *C_k_* in another branch, their vertical-overlap ratio is defined as follows:

(22) $$O_{y}\left(C_{r},C_{k}\right)=\frac{\left|Y_{r}\cap Y_{k}\right|}{\min\left(\left|Y_{r}\right|,\left|Y_{k}\right|\right)},$$

where *Y_r_* and *Y_k_* are their vertical ranges. The two components are regarded as detections of the same defect when their vertical-overlap ratio is at least 90% and the distance between their horizontal centers does not exceed 2*d*_max_, where *d*_max_ is the maximum permitted displacement defined in Section 2.4.2. Because the displacement of each branch is bounded by *d*_max_, the maximum permitted relative displacement between two branches is set to 2*d*_max_. If several components satisfy these conditions, the component with the smallest horizontal-center distance is selected.

## 3. Experimental Evaluation and Results

Using images acquired from an actual production line, this section evaluates the proposed method through comparisons of registration and local correction, assessments of reference-template strategies, and ablation and parameter sensitivity analyses. Three questions are addressed: (1) what characterizes the directionality of residual displacement and the practical extent of column-band compensation, and how do global registration alone, DIS dense optical flow [18], SIFT–Active Demons [17], and the proposed method compare in residual reduction, defect-response preservation, and final detection performance; (2) how does the proposed template-library strategy compare with a fixed single template and the multi-template edge-feature fusion method in ref. [7] in terms of detection performance and computational cost; and (3) how do template tolerance and multi-template voting individually affect the detection results, and how does detection performance vary with the main operating parameters.

### 3.1. Dataset and Evaluation Protocol

#### 3.1.1. Data Acquisition and Dataset Composition

All images were acquired from printed laminated film under actual industrial production conditions using the line-scan inspection platform described in Section 2.1. Each image had a resolution of 1225 pixels in width and 2048 pixels in height, corresponding to a physical imaging area of approximately 120 mm in width and 200 mm in height. A total of 5000 images of qualified products were selected from different production batches at a laminated-film factory. These images formed the qualified-reference candidate pool, from which 200 representative images were selected using the ORB–RANSAC-based farthest-first strategy described in Section 2.3.1 to construct the final normal template library.

The independent test set contained 1500 images randomly sampled from multiple production batches that were distinct from those used to construct the qualified-reference candidate pool, including 200 defect-bearing images and 1300 defect-free images. The defect status of each test image was confirmed by factory inspectors. Each visible defect was subsequently delineated using a separate pixel-level mask and assigned a unique instance identifier, resulting in 823 annotated defect instances. If one image contained several spatially separated defects, each defect was annotated and evaluated independently.

To ensure an independent evaluation, the qualified-reference candidate pool, the final template library, and the test set were strictly separated: no candidate-reference or template image appeared in the test set, and the reference and test data were collected from different production batches. For the main performance comparisons, a fixed parameter configuration was used for the proposed method across all test images. Once specified, the configuration of each comparison method was kept unchanged across the complete test set. The composition and experimental roles of the reference and test data are summarized in Table 2.

#### 3.1.2. Comparison Protocols and Fairness Controls

Two comparison groups were designed. The registration experiment kept the proposed reference strategy fixed. DIS changed only the local correction after ORB–RANSAC, whereas SIFT–Active Demons was included as an external complete registration pipeline. In addition to residual and visual comparisons, the three registration-correction pipelines were evaluated in terms of final defect-detection performance using the same downstream Frangi comparison and decision rules. The subsequent reference-template experiment compared a fixed single template, the published multi-template edge-feature fusion pipeline in [7], and the proposed template-library system. The fixed-template and proposed configurations used the same registration and detection settings, whereas ref. [7] was reproduced with its published edge-feature detection procedure.

The registration comparison included five configurations. ORB–RANSAC global only retained one homography without local correction. ORB–RANSAC + DIS applied DIS dense optical flow [18] after the same homography. SIFT global only used SIFT correspondences and RANSAC affine registration without elastic correction. SIFT–Active Demons reproduced the printed-image registration pipeline in [17] by adding Active Demons correction after the SIFT initialization. Proposed combined ORB–RANSAC with the residual-triggered, direction-constrained column-band compensation described in Section 2.4. All five configurations used the same qualified-template library and the complete independent test set listed in Table 2. The registration configurations are summarized in Table 3.

#### 3.1.3. Evaluation Metrics

Let ${\left\{G_{q}\right\}}_{q=1}^{N_{D}}$ denote the annotated instance masks and {*P_j_*} represent the connected components extracted from the final predicted mask. Each overlapping prediction is associated with only the annotated instance having the largest intersection, and ${\tilde{P}}_{q}$ denotes the union of the predictions associated with *G_q_*. The ground-truth coverage is defined as follows:

(23) $$C_{q}=\frac{\left|{\tilde{P}}_{q}\cap G_{q}\right|}{\left|G_{q}\right|}$$

Here, *C_q_* is the ground-truth coverage ratio, defined as the proportion of annotated defect pixels covered by the associated prediction. A threshold of 0.80 is used for the primary evaluation, requiring the prediction to cover at least 80% of the annotated defect area. The relatively high 80% threshold provides a stricter criterion for determining whether a small defect has been sufficiently detected, preventing a prediction that covers only a small portion of the defect from being counted as a successful detection. Each pixel corresponds to approximately 0.009566 mm^2^, and the minimum defect area specified for detection in this product is 0.04 mm^2^, equivalent to approximately 4.18 pixel areas. For an annotation containing five pixels, the coverage criterion requires at least four annotated pixels to be covered. An annotated defect is counted as a true positive *TP* when *C_q_* ≥ 0.80; otherwise, it is counted as a false negative *FN*. Predictions not contributing to a true-positive defect are counted as false positives *FP*. Instance recall and instance precision are calculated as follows:

(24) $$\mathrm{Recall}=\frac{\mathrm{TP}}{\mathrm{TP}+\mathrm{FN}}\times 100\%$$

(25) $$\mathrm{Precision}=\frac{\mathrm{TP}}{\mathrm{TP}+\mathrm{FP}}\times 100\%$$

To quantify sampling uncertainty while accounting for the dependence among multiple defect instances that co-occur within the same image, 95% confidence intervals for instance recall and instance precision were estimated using a stratified image-level bootstrap resampling strategy.

In each of 3000 replicates, 200 defect-bearing images and 1300 defect-free images were sampled separately with replacement from their corresponding subsets. All annotated instances and predicted components belonging to a sampled image were retained together, after which *TP*, *FP*, *FN*, and $N_{N}^{\mathrm{alarm}}$ were recomputed. The same resampled image indices were used for all compared methods to preserve paired comparisons. The 2.5th and 97.5th percentiles of each bootstrap distribution were used as the limits of the corresponding 95% confidence interval. The random seed was fixed at 2026.

To evaluate registration independently from the anomaly threshold, the mean absolute residual was calculated on every image in the independent test set. For a template *T_i_* and its registered or corrected inspected image $I_{i}^{*}$, the valid-region residual is given by:

(26) $$E_{i}=\frac{1}{\left|\Omega_{i}\right|}\sum_{\left(x,y\right)\in \Omega_{i}}\left|T_{i}\left(x,y\right)-I_{i}^{*}\left(x,y\right)\right|$$

Here, Ω*_i_* excludes invalid borders introduced by image warping. In the laminated-film images used in this experiment, the total annotated defect area in each defect-bearing image did not exceed 0.05% of that image’s area; therefore, defect pixels within *Ω_i_* were retained when calculating the mean absolute residual. Algorithm processing time was defined as the mean elapsed time per image from input of an inspection image to output of the results of the corresponding algorithmic procedure, excluding image acquisition, transfer and disk input/output.

For the defect-preservation analysis, the mean absolute residual inside each transformed ground-truth defect mask was measured before and after local correction. The defect-response retention ratio was defined as the post-correction response divided by the corresponding global-registration response. For instances with a positive pre-correction mean absolute residual, retention ratios below 0.90, 0.80, and 0.70 were counted as attenuation greater than 10%, 20%, and 30%, respectively, with cumulative counts reported at each threshold. Instances with zero pre-correction mean absolute residual were excluded from the attenuation-ratio analysis, and their number was reported separately. ORB-RANSAC global registration was used as the pre-correction baseline for DIS and the proposed method, whereas SIFT global registration was used for SIFT-Active Demons.

### 3.2. Implementation Details

The method was implemented in Python (3.9.1) using OpenCV (4.13.0.92) and NumPy (2.0.2) on a workstation equipped with an Intel Core i5-14600F CPU, 16 GB RAM, and Windows 11. All experiments were executed on the CPU using the parameter settings in Table 4. The remaining implementation parameters, software versions, and comparison-method configurations are provided in Zenodo at https://doi.org/10.5281/zenodo.22304265.

### 3.3. Comparison of Registration and Local Correction

#### 3.3.1. Residual-Displacement Directionality After Global Homography

To validate the assumption that residual deformation is primarily confined to column bands and occurs along the web-motion direction, 500 defect-free images were selected from the independent test set and analyzed after global homography and histogram matching but before local compensation. For each image, the highest-ranked branch after refined registration was used as the reference coordinate system. Candidate bands were localized using the same residual-triggered procedure as the proposed method. Within each band, control points were placed at intervals of 24 px, and a 41-px-wide local window centered at each control point was evaluated. The one-dimensional displacement search in Equation (10) was then extended to a symmetric two-dimensional translation search with the same bound *d*_max_ = 8 px along both image axes. For every candidate pair (*u*,*v*), the local mean absolute residual *E_c_*(*u*,*v*) was calculated as follows, and the values of *u* and *v* that produced the minimum *E_c_*(*u*,*v*) were taken as the horizontal and vertical residual estimates ${\hat{d}}_{x,c}$ and ${\hat{d}}_{y,c}$, respectively:

(27) $$E_{c}\left(u,v\right)=\frac{1}{\left|\Omega_{c}^{u,v}\right|}\sum_{\left(x,y\right)\in \Omega_{c}^{u,v}}\left|T_{r}\left(x,y\right)-{\tilde{I}}_{r}\left(x-u,y-v\right)\right|$$

where *u* and *v* are the candidate horizontal and vertical displacements, respectively; and $\Omega_{c}^{u,v}$ denotes the valid overlapping pixels of local window Ω*_c_* under translation (*u*,*v*). The discrete minimum of *E_c_*(*u*,*v*) was refined to subpixel precision by parabolic interpolation along both axes.

Table 5 shows that horizontal displacement exceeds 0.5, 1, and 3 px at 64.84%, 26.37%, and 5.49% of the sampled locations, respectively, compared to 15.38%, 1.10%, and 0.00% for vertical displacement. Together with the observed spatial distribution, this directional difference supports the hypothesis that residual deformation is largely confined to column-band regions and occurs predominantly along the direction of strip motion.

#### 3.3.2. Extent of Column-Band Compensation

To characterize the practical extent of column-band compensation, the numbers and widths of candidate and finally accepted bands were summarized over a reproducible random sample of 200 images from the independent test set (random seed: 20260920). Because three template branches were retained for each inspected image, the band counts for an image were obtained by summing the counts over its valid retained branches, whereas the width statistics were calculated over all corresponding bands. Images containing no candidate band or no accepted band were retained when calculating the count statistics. Table 6 reports the median, interquartile range, and observed range.

The 200 images yielded 1763 candidate bands and 1389 accepted bands over 600 valid template branches; no branch failed registration.

#### 3.3.3. Mean Absolute Registration Residual

The first experiment compared the five registration configurations defined in Section 3.1.2. For each configuration, the image-level absolute residual *E_i_* defined in Equation (26) was calculated on all *N*_test_ = 1500 images in the independent test set, and the dataset mean was obtained as follows:

(28) $$\bar{E}=\frac{1}{N_{\mathrm{test}}}\sum_{i=1}^{N_{\mathrm{test}}}E_{i}$$

The same 1500 images and the valid-region protocol defined in Section 3.1.3 were used for every configuration. Runtime was measured for the registration and method-specific local-correction stages after fixing the reference template; image loading, template retrieval, residual calculation, visualization, and disk output were excluded.

Table 7 directly compares the photometric agreement achieved by the five registration configurations. Relative to ORB–RANSAC global registration alone, the proposed column-band compensation reduces $\bar{E}\;$ by 35.33%, from 6.373424 to 4.121492. DIS and SIFT–Active Demons achieve still lower residuals of 3.728818 and 3.645880, respectively, whereas SIFT–Active Demons requires 2644.17 ms per image, which is approximately 9.86 times the registration-stage runtime of the proposed method. These results show that flexible dense deformation can further improve photometric agreement, but $\bar{E}$ alone does not establish suitability for defect inspection because it does not distinguish between corrected normal misalignment and attenuated defect responses. Detection performance is therefore evaluated separately in Section 3.3.5.

**Figure 5:**
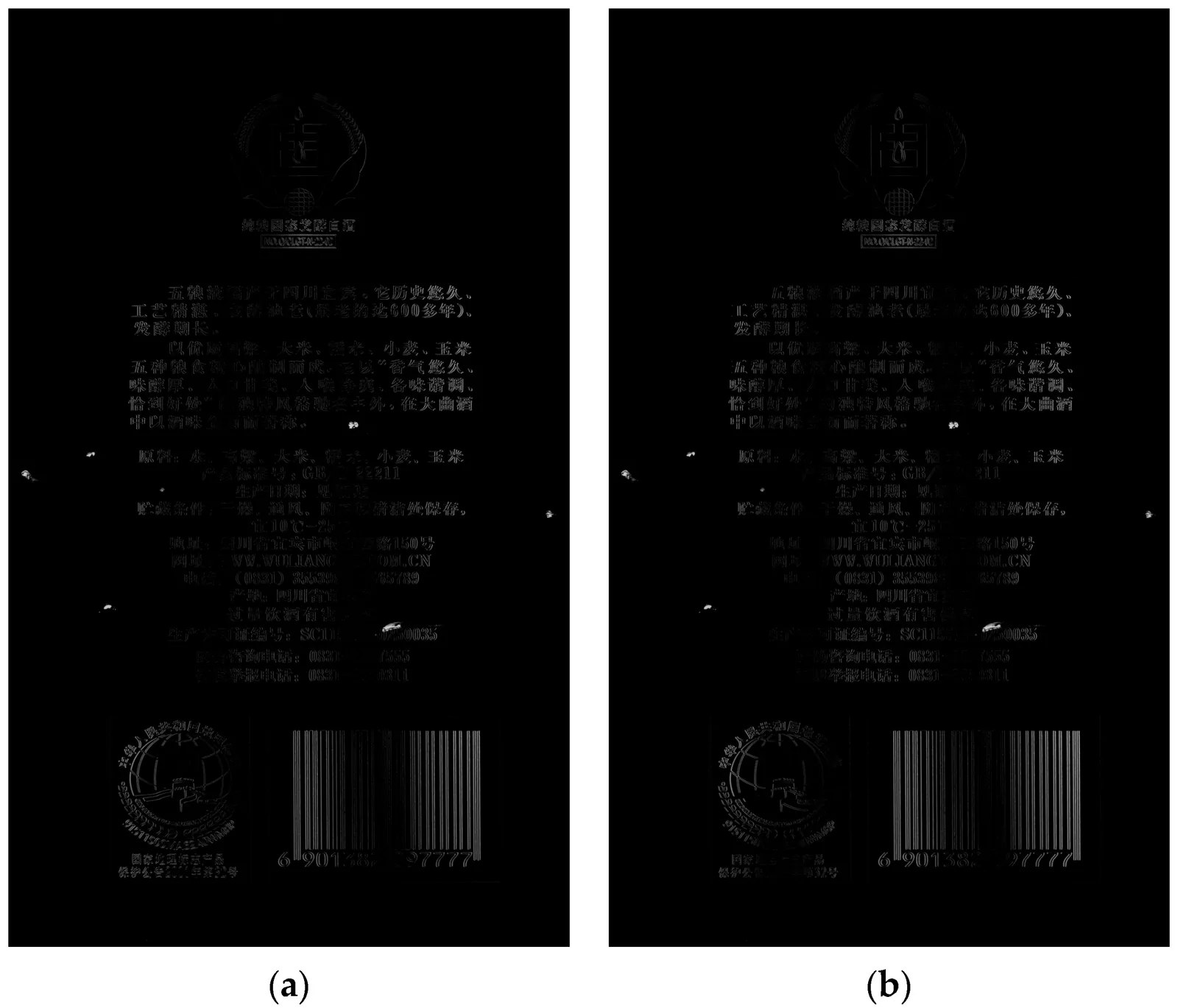
Absolute residual maps obtained using two global-registration schemes: (**a**) ORB–RANSAC homography used in the proposed pipeline; and (**b**) SIFT–RANSAC affine registration used to initialize SIFT–Active Demons. The Chinese characters visible in the images are part of the original printed product artwork and are not figure annotations.

Figure 5 shows that both global schemes remove most of the overall pose discrepancy between the inspected image and its reference, but structure-aligned residuals remain around printed text, logos, barcode edges, and lateral blocks. These responses arise mainly from spatially nonuniform stretching during line-scan imaging and cannot be fully represented by one global transformation.

#### 3.3.4. Visual and Quantitative Comparison of Deformation Models and Defect Preservation

**Figure 6:**
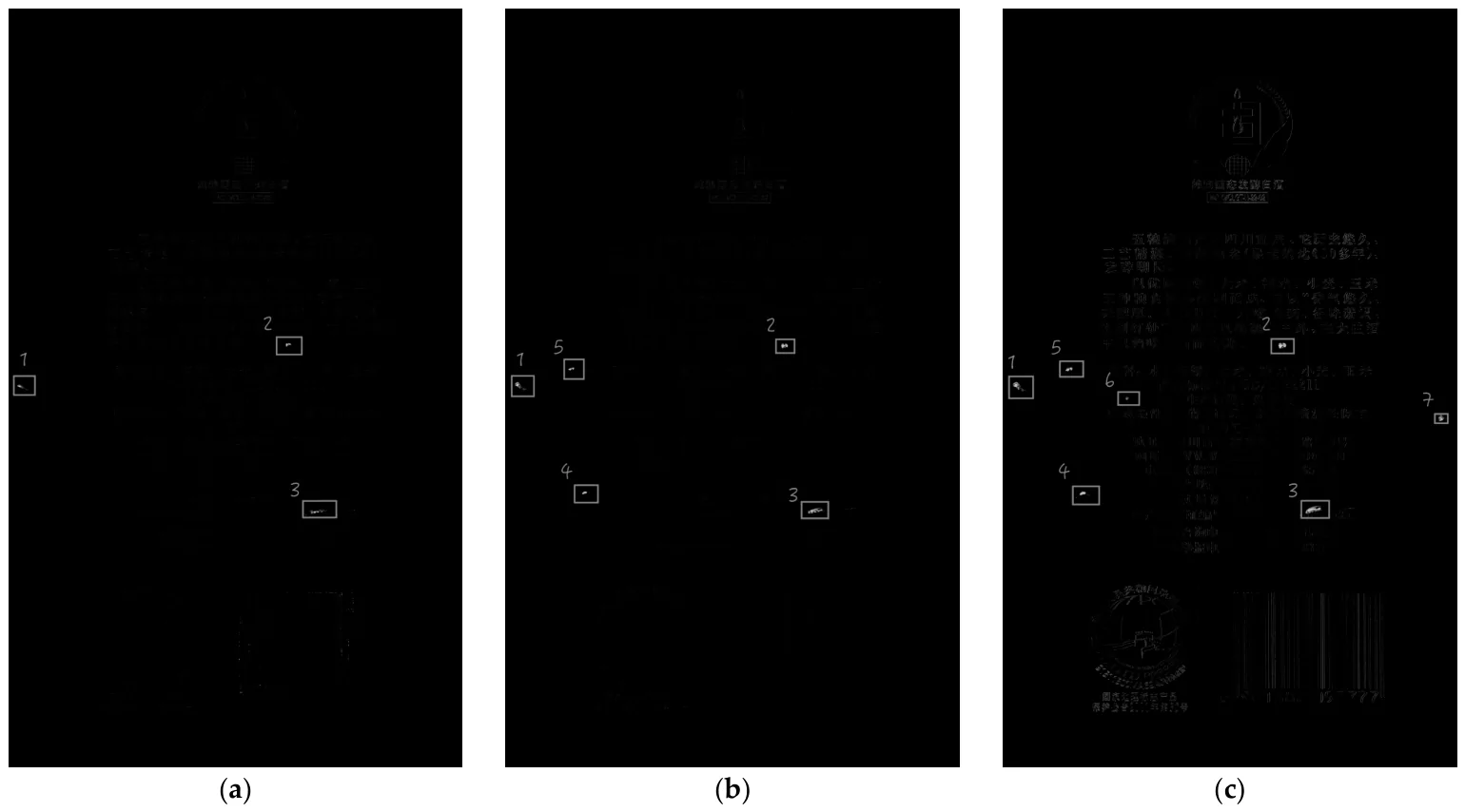
Defect-preservation comparison of three local-correction methods: (**a**) ORB–RANSAC followed by DIS dense optical flow; (**b**) SIFT–Active Demons; and (**c**) the proposed ORB–RANSAC followed by column-band compensation. Numbered boxes identify the defect responses that remain visible in each residual map. The Chinese characters visible in the images are part of the original printed product artwork and are not figure annotations.

The mean absolute residual alone cannot determine whether a registration method is suitable for defect inspection, because a highly flexible deformation model may reduce both misregistration residuals and true anomaly responses. Figure 6 therefore compares the residual maps of the three local-correction methods on the same defect-bearing image. The image, reference template, valid region, grayscale mapping, and display settings are kept identical. The numbered boxes indicate defect responses that remain visually distinguishable after each correction.

DIS retains three clearly visible marked defect responses and SIFT–Active Demons retains five, whereas the proposed method retains all seven in this example. The observation illustrates that stronger residual suppression may also weaken localized defects, but this single image does not establish dataset-wide detection rates. To complement the representative case in Figure 6, a quantitative defect-preservation analysis was designed for the 823 annotated defect instances, counting defects with response attenuation greater than 10%, 20%, and 30% after local correction, as reported in Table 8.

**Figure 7:**
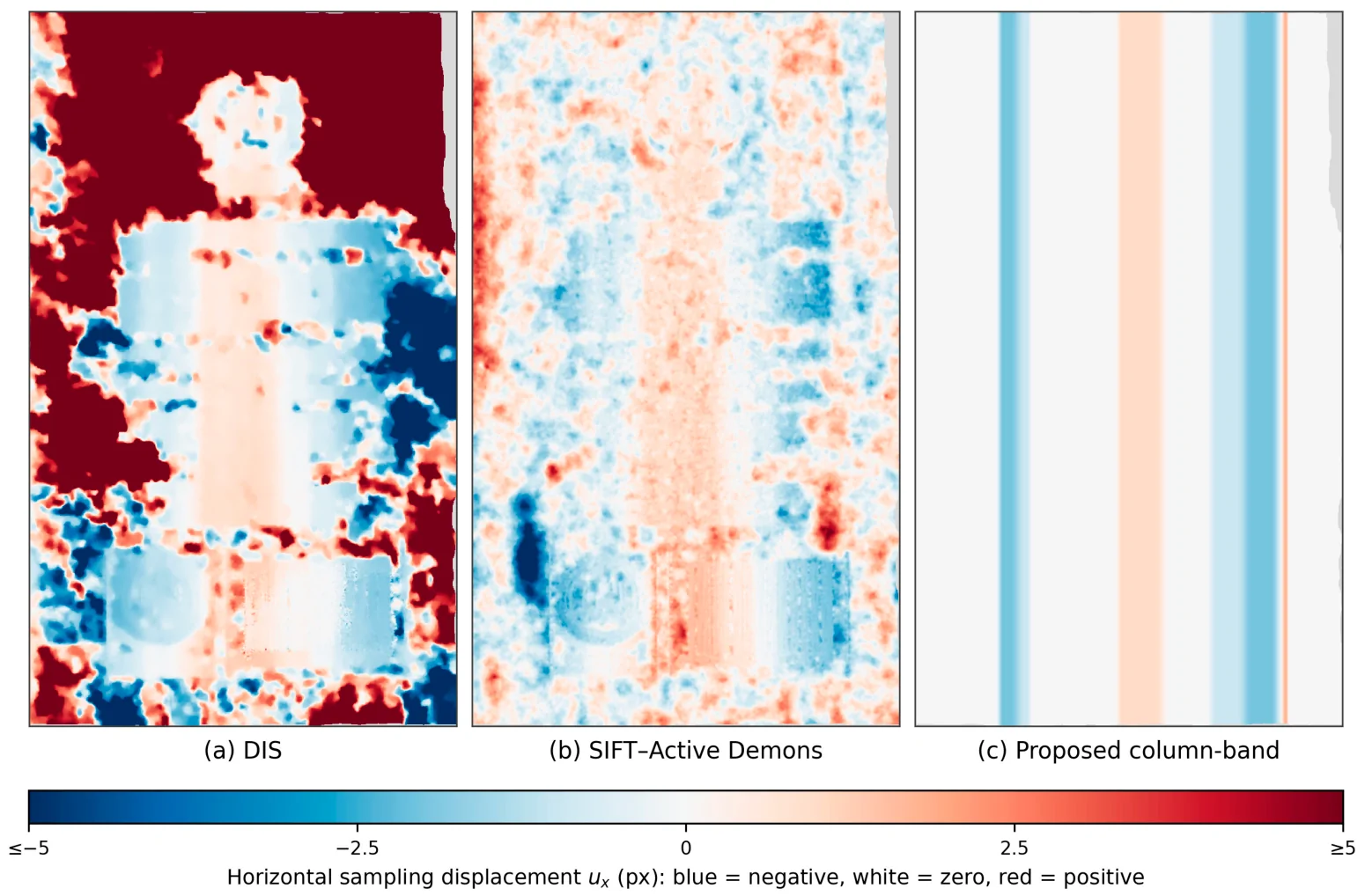
Horizontal sampling-displacement fields of (**a**) DIS dense optical flow, (**b**) SIFT–Active Demons, and (**c**) the proposed column-band compensation. All panels show *u_x_* in pixels using a shared [−5, 5] px color scale; values outside this range are clipped for visualization. Blue, white, and red indicate negative, zero, and positive sampling displacement, respectively.

At attenuation thresholds above 10%, 20%, and 30%, the proposed method records 60, 26, and 11 instances, respectively, compared to 270, 177, and 112 instances for DIS and 202, 126, and 64 instances for SIFT–Active Demons. The reported counts indicate fewer instances with pronounced response attenuation under the proposed correction. Figure 7 further compares the horizontal sampling-displacement component *u_x_* generated by the three correction methods. All panels use the same physical unit and color scale: blue denotes negative displacement, white denotes zero displacement, and red denotes positive displacement. Values outside [−5, 5] px are clipped only for visualization.

#### 3.3.5. Final Defect-Detection Performance

To quantify how the registration-correction model affects the final inspection decision, DIS, SIFT–Active Demons, and the proposed column-band compensation were evaluated using the same independent test set and the instance-level protocol defined in Section 3.1.3. After each registration pipeline, the same Frangi comparison, multi-template voting, and component-screening rules were applied. Thus, Table 9 complements the residual results in Table 7 by measuring whether residual suppression is achieved without sacrificing defect responses.

The proposed pipeline achieves a recall of 92.95%, exceeding DIS and SIFT–Active Demons by 13.61 and 7.90 percentage points, respectively, while maintaining a precision of 96.11%. Its recall 95% confidence interval (91.02–94.75%) lies entirely above those of DIS (75.77–82.72%) and SIFT–Active Demons (81.94–87.81%), further supporting the observed recall advantage. These results support the effectiveness of the proposed pipeline in retaining defect sensitivity while maintaining high precision.

To examine detection performance across defect sizes, the annotated pixel area *A_q_* = |*G_q_*| was used as the size measure. Instances with 5 ≤ *A_q_* ≤ 15, 15 < *A_q_* ≤ 40, and *A_q_* > 40 px were categorized as small-area, medium-area, and large-area defects, respectively. Using an approximate physical area of 0.009566 mm^2^ per pixel, the physical defect area is $A_{q}^{\mathrm{phys}}\approx 0.009566A_{q}$ mm^2^, giving group boundaries of approximately 0.047830, 0.143490, and 0.382640 mm^2^.

Table 10 shows that the recall advantage is most pronounced for small defects, for which the proposed method achieves 91.00%, exceeding DIS and SIFT–Active Demons by 23.50 and 13.50 percentage points, respectively. The corresponding gains are 5.72 and 3.58 percentage points for medium defects, but only 1.39 and 0.70 percentage points for large defects. This pattern suggests that the proposed method better preserves the detectability of small and medium defects, with the greatest benefit observed for small defects, which account for 400 of the 823 instances (48.60%). Local compensation driven by absolute-residual minimization in DIS and SIFT–Active Demons can also suppress defect-related differences, particularly for small defects. The proposed compensation strategy may better retain these differences, thereby reducing missed detections.

### 3.4. Comparison of Reference-Template Strategies

The fixed single-template baseline uses one qualified reference selected before evaluation and applies the same ORB–RANSAC registration, column-band compensation, and Frangi comparison to every inspected image. The multi-template edge-feature fusion baseline follows Liu et al. [7], in which registered qualified images are divided into sub-blocks and converted into a fused Canny-edge representation for similarity matching. The proposed strategy retains the top *K* geometrically compatible templates as separate complete references, processes them independently, and combines their anomaly masks by voting.

The fixed-template and proposed configurations share the same registration, Frangi, and component-screening settings, so their difference directly reflects the use of multiple complete references and voting. The method in ref. [7] is treated as an external multi-template baseline and is reproduced using its published feature-extraction and matching procedure; therefore, it is not interpreted as a single-module ablation. All three strategies use the same independent test set and evaluation protocol.

**Figure 8:**
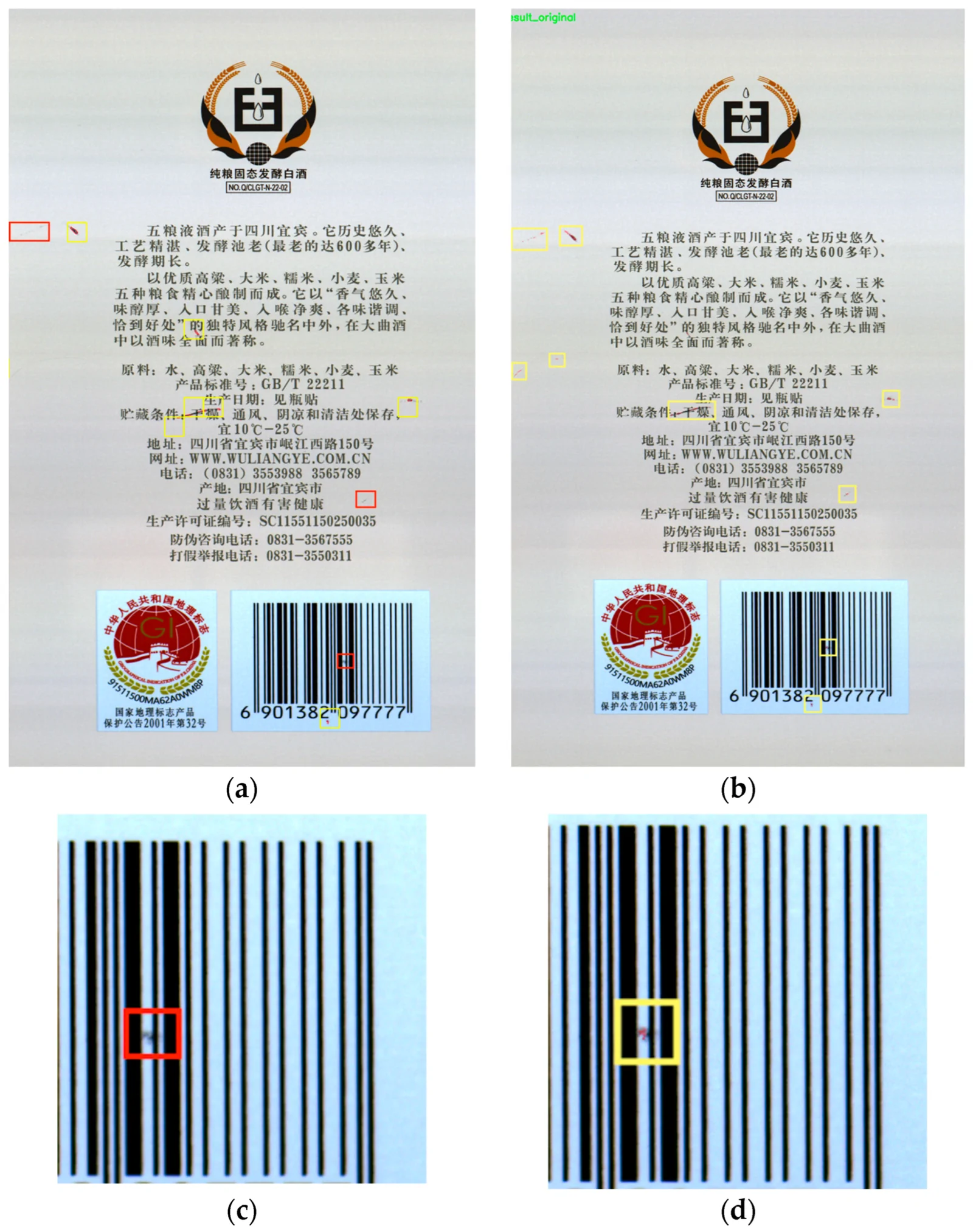
Qualitative comparison of reference-template strategies: (**a**) detection result of multi-template edge-feature fusion; (**b**) detection result of the proposed full-reference template library; (**c**) enlarged barcode region from (**a**), where the red box marks a missed defect; and (**d**) enlarged barcode region from (**b**), where the yellow box indicates the detected defect. Yellow boxes denote detector outputs, whereas red boxes are manual annotations of missed defects and are not detector outputs.

Table 11 shows that relative to a fixed template, the proposed library increases recall by 23.69 percentage points and precision by 25.83 points. Compared to edge-feature fusion, it raises recall by 4.86 points while reducing precision by 2.00 points and increasing processing time from 135.24 to 357.06 ms/image (2.64-fold). Relative to the multi-template edge-feature fusion method, the proposed system achieves higher recall but lower precision and longer processing time. The multi-template edge-feature fusion method [7] shows limited sensitivity to defects in regions with rich textures or densely arranged patterns. As illustrated in Figure 8, it fails to detect defects within the dense barcode region. This limitation may arise from its reliance on local edge information without sufficient constraints on the overall image structure, making it difficult to distinguish defect-induced differences from those caused by image displacement in densely patterned regions. The library strategy therefore improves defect sensitivity at an additional computational cost.

### 3.5. Ablation and Parameter Sensitivity Analysis

#### 3.5.1. Ablation of Template Tolerance and Multi-Template Voting

The third experiment isolated the two decision-stage mechanisms after registration and column-band compensation. A two-factor ablation was used: template tolerance was either enabled or disabled, and the output was produced either by all *K* masks or by the first retained candidate alone. The candidate set, registration results, Frangi scales, anomaly threshold, and component-screening criteria remained unchanged. This 2 × 2 design separated the effect of tolerance around normal printed structures from the effect of consensus among independent templates.

In Table 12, a check mark (✓) indicates that the corresponding component is enabled, whereas a blank cell indicates that it is disabled. Table 12 shows that enabling template tolerance alone increases precision from 70.48% to 84.67%, with recall decreasing by 0.36 percentage points. Voting alone raises precision to 86.43% without changing the displayed recall. Enabling both yields 96.11% precision and 92.95% recall, representing a precision gain of 25.63 points at a recall cost of 0.73 points relative to disabling both. Both components contribute to reducing false-positive predictions, while their combination provides the highest precision among the four configurations; the combined configuration nevertheless incurs a recall cost.

#### 3.5.2. Effect of the Main Operating Parameters

**Figure 9:**
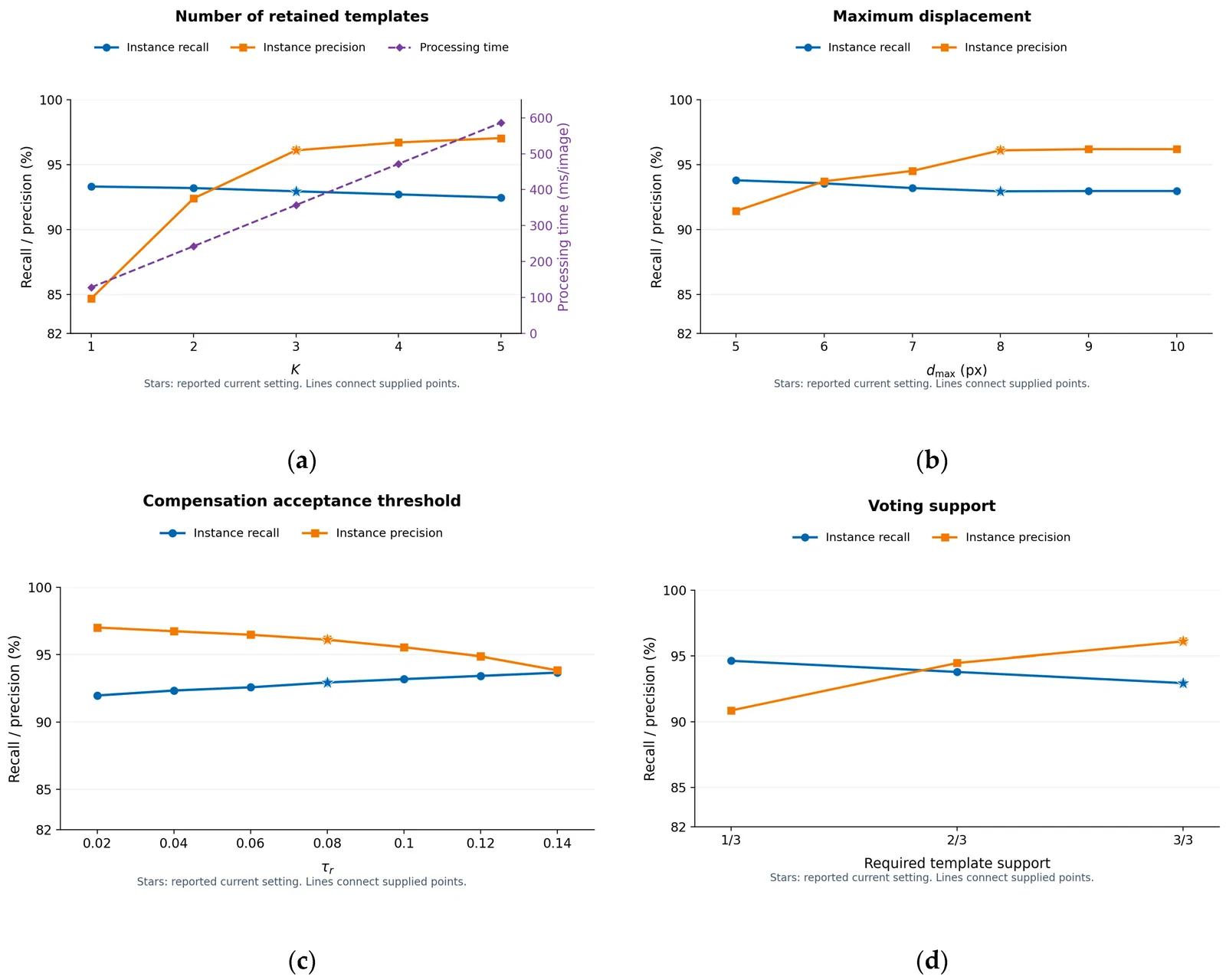
Effects of the main operating parameters on instance recall and instance precision: (**a**) number of retained templates *K*, with processing time additionally shown on the right axis; (**b**) displacement limit *d*_max_; (**c**) compensation acceptance threshold *τ_r_*; and (**d**) voting support at fixed *K* = 3. Stars indicate the current configuration.

The sensitivity-analysis design considered four operating parameters: the number of retained templates *K* ∈ 1,2,3,4,5, the displacement limit *d*_max_ ∈ {5,6,7,8,9,10} px, the compensation acceptance threshold *τ_r_* ∈ {0.02,0.04,0.06,0.08,0.10,0.12,0.14}, and voting support of 1/3, 2/3, or 3/3 at fixed *K* = 3. The protocol varied one parameter at a time with the other settings fixed at their baseline values. For the template-count comparison, unanimous *K*/*K* voting was retained, so the comparison included the accompanying change in consensus requirements. Figure 9 presents the experimental results for the four parameters.

In Figure 9a, increasing *K* from one to three raises precision from 84.67% to 96.11%, while recall decreases from 93.32% to 92.95%. Increasing *K* further to five adds only 0.94 percentage points of precision, reduces recall by another 0.48 points, and raises processing time from 357.06 to 586.28 ms/image. These diminishing precision gains support *K* = 3 as a practical compromise. In Figure 9b, increasing the displacement limit from 5 to 8 px improves precision from 91.43% to 96.11%, while recall decreases slightly from 93.80% to 92.95%. Both metrics remain unchanged as the limit increases further to 9–10 px, indicating that expanding the compensation range beyond 8 px provides no additional detection benefit. Therefore, 8 px is selected as the smallest tested displacement limit that achieves plateau performance. In Figure 9c, increasing the acceptance threshold from 0.02 to 0.14 raises recall from 91.98% to 93.68% but lowers precision from 97.02% to 93.85%. This trend is consistent with accepting fewer corrections and retaining more candidate responses, including both defects and false positives. The current threshold of 0.08 provides an intermediate operating point, without simultaneously maximizing both metrics. Figure 9d shows that moving from 1/3 to 3/3 voting increases precision by 5.25 percentage points, from 90.86% to 96.11%, while reducing recall by 1.70 points. Unanimous voting therefore suppresses unsupported predictions but can also remove true defects. The choice of 3/3 favors precision relative to the other tested voting rules and should be interpreted as a task-dependent trade-off.

Image acquisition and processing operate asynchronously. On a computer equipped with an Intel Core i5-14600F processor and 16 GB of RAM, the mean processing time was 357.06 ms per image, including defect localization and generation of visual annotations. This corresponds to a sequential processing capacity of approximately 2.80 images/s. Because each image covers 120 mm along the web-motion direction, this capacity is equivalent to an estimated continuous line speed of approximately 20.16 m/min, assuming no gap or overlap between consecutive images. It exceeds the normal acquisition rate of 2 images/s (14.4 m/min) but remains below the maximum acquisition rate of 5 images/s (36.0 m/min). The current implementation therefore supports sustained operation at the normal production speed, whereas operation at the maximum speed requires further acceleration or increased processing concurrency. The present conclusions should therefore be restricted to production conditions represented by the evaluated library, test batches, imaging configuration, and deformation model.

## 4. Discussion

Constraining local correction reduces the risk of absorbing a defect but does not remove it. In Table 8, the proposed correction attenuates the residual response by more than 10%, 20%, and 30% for 60, 26, and 11 of the 823 annotated instances, respectively. At a regular rectangular boundary, printing-layer misregistration may resemble horizontal imaging drift; if the defect lies in an accepted band, compensation may weaken its response. A second failure route occurs during cross-template voting: a weak or slightly misaligned defect that is retained in only one or two branches is removed by the 3/3 rule. The reference and test images in this study were collected from different production batches using the same production line and the same industrial line-scan camera system. The imaging drift along the moving direction is mainly caused by fluctuating film tension and uneven material motion. Therefore, the current evaluation is only valid for specific industrial scenarios where images are captured by industrial line-scan cameras and motion-direction drift is induced by unstable film tension and irregular transportation motion. The experimental results cannot confirm the generalization and robustness of the proposed method under varying film structures, camera devices, illumination configurations, or web-tension conditions.

## 5. Conclusions

This study presents an interpretable machine-vision system for line-scan inspection of printed laminated films under appearance variation and spatially nonuniform mismatch. A qualified multi-template library and coarse-to-fine matching establish global correspondence through low-resolution screening, intermediate-resolution ORB-RANSAC registration, and original-resolution warping. Residual-driven column-band compensation then corrects only residual-selected bands and constrains displacement to the web-motion direction. Template-tolerant multiscale Frangi comparison and component-level consensus voting further suppress responses caused by residual misregistration. On the independent production-line test set, the system achieved 92.95% instance recall and 96.11% instance precision. Its recall exceeded that of the two dense-registration alternatives and multi-template edge-feature fusion [7]. Relative to ref. [7], this recall gain was accompanied by a 2.00-percentage-point decrease in precision and a 2.64-fold increase in processing time, representing a task-dependent trade-off in favor of defect sensitivity. The qualitative comparison showed that the constrained correction retained more visible defect responses than the dense methods, while the ablation study confirmed the complementary effects of template tolerance and multi-template voting. These findings demonstrate an effective balance between mismatch compensation and defect preservation under the evaluated production conditions.

This effectiveness is limited to multiple batches acquired from one production line using the same imaging platform. Robustness across different products, materials, imaging conditions, web-tension regimes, and production speeds, as well as stable long-term deployment, remains unverified. Independently processing multiple references increases computational cost, and the system still depends on engineering-selected parameters and adequate template-library coverage. Its measured capacity of 2.80 images/s (20.16 m/min) supports the evaluated normal speed of 14.4 m/min but not sustained operation at 36.0 m/min. Defects within accepted bands or retained by fewer than all template branches may also be weakened or missed. Future work will examine broader operating conditions, adaptive parameter and template-library updating, and parallel implementation.

## Figures and Tables

**Table 1:** Main components of the inspection system.

Component	Model	Function
Line-scan camera	MV-CL024-91GC (Hangzhou Hikrobot Co., Ltd., Hangzhou, China)	Line-by-line acquisition of the printed web
Roll-to-roll unit	LTD automatic rewinder (Tmaxmachine, Xiamen, China)	Stable transport of the printed laminated film
Color-mark photoelectric sensor	SR21-RG (Datasensing S.r.l., Modena, Italy)	Frame-level trigger at each repeated printed unit
Encoder	ZL38S6-500-SM5-23C-2M (OMRON, Shanghai, China)	Line trigger synchronized with web motion
Line-light illumination	MV-LTDS-300-W (Hangzhou Hikrobot Co., Ltd., Hangzhou, China)	Uniform illumination of the imaging region
Edge-guiding device	TH35-35020080B-R-H-D (Techmach, Beijing, China)	Suppression of lateral web wandering
Industrial computer	MIC-770 (Advantech Co., Ltd., Taipei, Taiwan)	Image acquisition, processing, display, and storage

**Table 2:** Composition and roles of the experimental data.

Data Subset	Role	Number of Images	Defect-Bearing Images	Annotated Defect Instances
Qualified-reference candidate pool	Source pool for template-library construction	5000	0	0
Final normal template library	Reference images used by the proposed system	200	0	0
Independent test set	Final performance evaluation	1500	200	823

**Table 3:** Configurations used for the registration comparison.

Method	Global Registration	Local Correction	Degrees of Freedom
ORB–RANSAC global only	ORB–RANSAC homography	None	One homography per image
ORB–RANSAC + DIS	ORB–RANSAC homography	DIS dense 2D flow	Two displacement values per pixel
SIFT global only	SIFT–RANSAC affine registration	None	One affine transformation per image
SIFT–Active Demons	SIFT-based global registration	Active Demons dense elastic correction	Dense 2D deformation field
Proposed	ORB–RANSAC homography	Residual-triggered horizontal column-band compensation	Bounded 1D displacement in selected bands

**Table 4:** Main implementation parameters.

Stage	Parameter	Value
Low-resolution screening	Image width/ORB features	256 px/700
	Number of retained templates *K*	3
Refined global registration	Image width/ORB features	512 px/2500
	Lowe ratio/RANSAC threshold	0.70/3.0 px
Column-band compensation	Smoothing window/band padding *w_p_*	21 px/24 px
	Localization parameters *τ*_0_/*κ*	6.0/0.70
	Displacement limit/acceptance threshold *d*_max_/*τ_r_*	8 px/0.08
Frangi comparison	Scales Σ	{0.5,0.7,1.0,1.5}
	Frangi parameters *β*/*c*	0.2/5.0
	Structural threshold *τ_F_*	0.07
	Tolerance scale/offset *α*/δ	1.2/0.02
	Upper/lower-bound kernel size	5 px
Component screening	Minimum area/excluded border	5 px^2^/10 px
Multi-template voting	Voting rule	Unanimous (3/3)
	Vertical-overlap threshold	0.9
	Horizontal-center tolerance	2*d*_max_ = 16 px

**Table 5:** Residual-displacement components measured on 500 defect-free images after global homography.

Component	>0.5 px	>1 px	>2 px	>3 px
Horizontal |*d_x_*|	64.84%	26.37%	6.59%	5.49%
Vertical |*d_y_*|	15.38%	1.10%	0.00%	0.00%

**Table 6:** Typical numbers and widths of candidate and accepted column bands in a 200-image random sample from the independent test set.

Band Statistic	Candidate Bands	Accepted Bands
Bands per inspected image, median [IQR]	9 [8–10]	7 [5.75–8]
Bands per inspected image, observed range	4–13	1–12
Band width, median [IQR] (px)	149 [97–196]	154 [105–195]
Band width, observed range (px)	49–632	49–632

**Table 7:** Mean absolute registration residuals on the independent test set.

Registration Configuration	Mean Absolute Residual $\bar{E}$	Runtime (ms/image)
Global registration only	6.373424	74.24
Global registration + DIS dense optical flow	3.728818	174.08
SIFT global registration only	7.329307	119.49
SIFT–Active Demons	3.645880	2644.17
Proposed global registration + column-band compensation	4.121492	268.06

**Table 8:** Defect-response attenuation after local correction.

Local-Correction Method	Defects Attenuated by >10% (Count)	Defects Attenuated by >20% (Count)	Defects Attenuated by >30% (Count)
ORB–RANSAC + DIS dense optical flow	270	177	112
SIFT–Active Demons	202	126	64
Proposed ORB–RANSAC + column-band compensation	60	26	11

**Table 9:** Final defect-detection performance of the three registration-correction pipelines on the independent test set.

Registration–Correction Pipeline	Instance Recall (%)	Recall 95% CI	Instance Precision (%)	Precision 95% CI
ORB–RANSAC + DIS dense optical flow	79.34	75.77–82.72	97.32	96.08–98.45
SIFT–Active Demons	85.05	81.94–87.81	97.22	96.04–98.32
Proposed ORB–RANSAC + column-band compensation	92.95	91.02–94.75	96.11	94.73–97.42

**Table 10:** Recall across defect-area groups.

Defect-Area Group	Instance Count	DIS Recall (%)	SIFT–Active Demons Recall (%)	Proposed Recall (%)
Small-area	400	67.50	77.50	91.00
Medium-area	280	88.57	90.71	94.29
Large-area	143	94.41	95.10	95.80

**Table 11:** Comparison of reference-template strategies on the independent test set.

Strategy	Instance Recall (%)	Recall 95% CI	Instance Precision (%)	Precision 95% CI	Algorithm Processing Time (ms/image)
Fixed single template	69.26	65.04–73.33	70.28	66.33–74.12	127.84
Multi-template edge-feature fusion [7]	88.09	85.21–90.75	98.11	96.85–99.17	135.24
Proposed template library	92.95	91.02–94.75	96.11	94.73–97.42	357.06

**Table 12:** Two-factor ablation of template tolerance and multi-template voting.

Template Tolerance	*K*-Template Voting	Instance Recall (%)	Instance Precision (%)
		93.68	70.48
✓		93.32	84.67
	✓	93.68	86.43
✓	✓	92.95	96.11

## Data Availability

The industrial production-line images and corresponding annotations are not publicly available because they contain commercially confidential product and packaging information. Requests to access the data should be directed to the corresponding author and will be considered subject to approval by the data owner.
